# Overexpression of the *Sorghum bicolor SbCCoAOMT* alters cell wall associated hydroxycinnamoyl groups

**DOI:** 10.1371/journal.pone.0204153

**Published:** 2018-10-05

**Authors:** Hannah M. Tetreault, Erin D. Scully, Tammy Gries, Nathan A. Palmer, Deanna L. Funnell-Harris, Lisa Baird, Javier Seravalli, Bruce S. Dien, Gautam Sarath, Thomas E. Clemente, Scott E. Sattler

**Affiliations:** 1 Wheat, Sorghum and Forage Research Unit, USDA-ARS, Lincoln, Nebraska, United States of America; 2 Department of Agronomy and Horticulture, University of Nebraska-Lincoln, Lincoln, Nebraska, United States of America; 3 Department of Plant Pathology, University of Nebraska-Lincoln, Lincoln, Nebraska, United States of America; 4 Department of Biology, Shiley Center for Science and Technology, University of San Diego, San Diego, California, United States of America; 5 Redox Biology Center and Department of Biochemistry, University of Nebraska-Lincoln, Lincoln, Nebraska, United States of America; 6 National Center for Agricultural Utilization Research, USDA-ARS, Peoria, Illinois, United States of America; 7 Center for Plant Science Innovation, University of Nebraska, Lincoln, Nebraska, United States of America; RIKEN Center for Sustainable Resource Science, JAPAN

## Abstract

Sorghum (*Sorghum bicolor*) is a drought tolerant crop, which is being developed as a bioenergy feedstock. The monolignol biosynthesis pathway is a major focus for altering the abundance and composition of lignin. Caffeoyl coenzyme-A *O*-methyltransferase (CCoAOMT) is an *S*-adenosyl methionine (SAM)-dependent *O*-methyltransferase that methylates caffeoyl-CoA to generate feruloyl-CoA, an intermediate required for the biosynthesis of both G- and S-lignin. *SbCCoAOMT* was overexpressed to assess the impact of increasing the amount of this enzyme on biomass composition. *SbCCoAOMT* overexpression increased both soluble and cell wall-bound (esterified) ferulic and sinapic acids, however lignin concentration and its composition (S/G ratio) remained unaffected. This increased deposition of hydroxycinnamic acids in these lines led to an increase in total energy content of the stover. In stalk and leaf midribs, the increased histochemical staining and autofluorescence in the cell walls of the *SbCCoAOMT* overexpression lines also indicate increased phenolic deposition within cell walls, which is consistent with the chemical analyses of soluble and wall-bound hydroxycinnamic acids. The growth and development of overexpression lines were similar to wild-type plants. Likewise, RNA-seq and metabolite profiling showed that global gene expression and metabolite levels in overexpression lines were also relatively similar to wild-type plants. Our results demonstrate that *SbCCoAOMT* overexpression significantly altered cell wall composition through increases in cell wall associated hydroxycinnamic acids without altering lignin concentration or affecting plant growth and development.

## Introduction

Sorghum (*Sorghum bicolor*) is a C_4_ grass being developed as a feedstock for conversion of biomass to biofuels. Indigenous to Africa, this crop exhibits high levels of drought tolerance and an ability to grow under low nutrient conditions, which allows it to be sustainably grown on marginal lands [1]. Sorghum is an ideal system for bioenergy research due its relatively small diploid genome (~730 Mb) and a wide range of genetic resources that includes a high quality reference genome [2]. Improving biomass yields and biomass composition are necessary to foster the replacement of petroleum-derived chemical precursors with those derived from lignocellulosic sources [3–5].

Cell walls, which are comprised of three main polymers cellulose, hemicellulose and lignin, are a major target for improving bioenergy conversion of sorghum biomass into biofuels and other renewable products [6, 7]. Lignin polymers are cross-linked to a hemicellulose network, and essential to the viability of land plants, however lignin impedes deconstruction of plant cell wall polysaccharides into fermentable sugars and substantially increases the costs of cellulosic ethanol production [6, 8, 9]. Therefore, considerable efforts have been directed toward altering lignin concentration and composition. Recently, there has been increasing interest in developing ways to valorize lignin for a range of applications [7, 10, 11].

**Fig 1:**
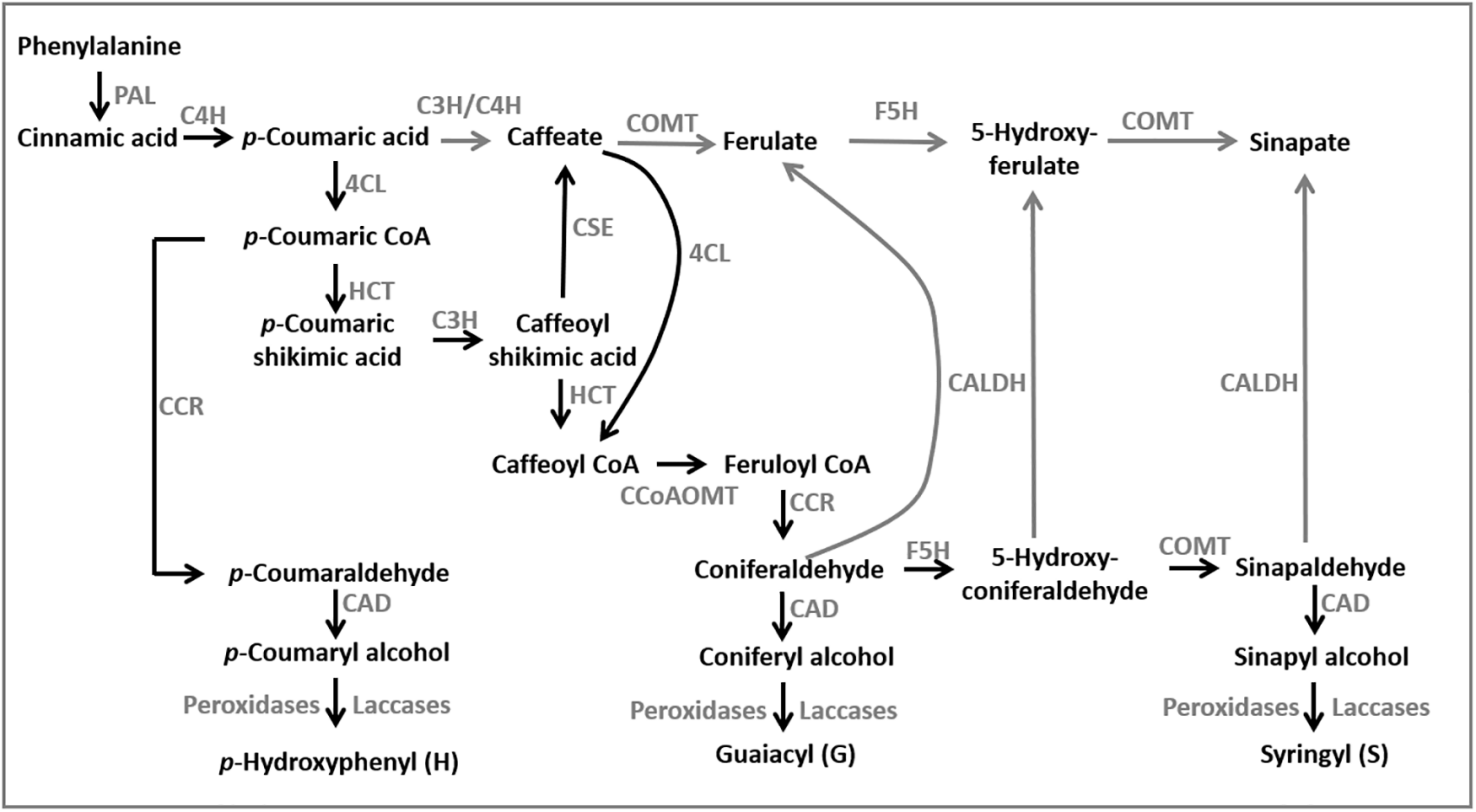
The monolignol biosynthesis pathway in sorghum based on consensus model from dicot and monocot plants (adapted from [25, 26]). Enzymes along the pathway (gray) represent: PAL, phenylalanine ammonia lyase; C4H, cinnamate 4-hydroxylase; 4CL, 4-coumarate-CoA ligase: HCT, *p*-hydroxycinnamoyltransferase; C3H, 4-coumarate hydroxylase; CSE, caffeoyl shikimate esterase; CCoAOMT, caffeoyl-CoA-O-methyltransferase; CCR, cinnamoyl-CoA reductase; F5H, ferulate 5-hydroxylase; COMT, caffeic acid O-methyltransferase; CALDH, cinnamyl aldehyde dehydrogenase CAD, cinnamyl alcohol dehydrogenase. Gray lines indicate proposed steps in the pathway.

The monolignol biosynthesis pathway is a major target for altering lignin content and composition, because this well-characterized and highly conserved pathway across vascular plants synthesizes the monomers of lignin polymers [12–14]. Lignin is polymerized through the oxidative radicalization of three major monolignols; *p*-coumaryl, coniferyl and sinapyl alcohols, which form *p-*hydroxyphenyl (H), guaiacyl (G) and syringyl (S) lignin subunits, respectively (Fig 1). Mutational, antisense and RNA interference (RNAi) approaches have been successfully used to impair the function of genes encoding monolignol biosynthetic enzymes, and have resulted in plants with reduced lignin content in several species [15–19]. In sorghum and other C_4_ grasses, the *brown midrib (bmr)* phenotype has been useful to identify a non-redundant set of mutants impaired in lignin synthesis [20]. Three sorghum *Bmr* genes have been characterized and shown to encode enzymes in monolignol biosynthesis; *Bmr2* (4-coumarate-CoA ligase, 4CL), *Bmr6* (cinnamyl alcohol dehydrogenase, CAD) and *Bmr12* (caffeic acid O-methyltransferase, COMT) [21–24].

In addition to COMT, the other *S*-adenosyl-L-methionine (SAM)-dependent *O-*methyltransferase, caffeoyl coenzyme-A *O*-methyltransferase (CCoAOMT) is involved in monolignol biosynthesis. This enzyme preferentially catalyzes the methylation of the 3-hydroxyl group of caffeoyl-CoA to generate feruloyl-CoA that is required for the synthesis of both G- and S-lignin (Fig 1) [27]. In contrast, COMT preferentially catalyzes the methylation of the 5-hydroxyl group of 5-hydroxyconiferaldehyde to produce sinapaldehyde in the synthesis of S-lignin. In some cases, these two methyltransferases may function interchangeably in the methylation of their preferred forms of caffeoyl or 5-hydroxyconiferoyl groups [28]. However, sorghum COMT can utilize caffeic acid, 5-hydroxyconiferaldehyde and 5-hydroxyconiferyl alcohol as substrates, but not caffeoyl alcohol or caffeoyl-CoA [29]. Furthermore, sorghum CCoAOMT displayed a strong preference for caffeoyl-CoA as a substrate, and did not efficiently bind nor has enzymatic activity for 5-hydroxyferuloyl-CoA or caffeic acid [30]. These studies indicate the function of COMT is in the synthesis of S-lignin, whereas the function of CCoAOMT is to synthesize feruloyl-CoA.

Feruloyl-CoA is a substrate for cinnamoyl-Coenzyme A reductase (CCR) of monolignol synthesis [31]. In addition, feruloyl-CoA is the substrate for ester-linked ferulates within cell walls [32–36], which are major features of grass cell walls together with other esterified hydroxycinnamic acid residues. Both ferulic and *p*-coumaric acids can be esterified to arabinose residues of glucuronoarabinoxylans (GAX) through the activity of BAHD acyltransferases using feruloyl-CoA and coumaroyl-CoA as substrates [36], which ultimately leads to cross-linking between GAX polymers, lignin and structural proteins in cell walls [37]. The creation of these cross-linkages is hypothesized to enhance defenses against pathogen invasion [38, 39]. Hydroxycinnamates including ferulic acid have been associated with inhibiting the growth of the pathogenic fungi *Fusarium* spp. in sorghum [40–43]. Ferulic acid is being proposed as a natural product for a range of applications including use as a natural food preservative to inhibit lipid peroxidation [44]. Ferulic acid is well recognized for its antioxidant activity [44–46], but also as a natural skin protectant against UV irradiation [47–49]. The ferulate fraction of biomass already has value for its potential commercial uses.

There have been several studies that have investigated the effects of decreasing *CCoAOMT* expression on lignin biosynthesis, and very few studies have examined effects of increasing *CCoAOMT* levels in plants. Decreasing *CCoAOMT* gene expression in tobacco produced dwarfed plants and an increase in lignin S/G ratio [27, 50] and decreased lignin content and increased S/G ratio in maize [51]. In the present study, *SbCCoAOMT* was overexpressed in sorghum to increase capacity of this step of phenylpropanoid metabolism and assess the potential impact on cell wall composition and plant health. Previously, overexpression of *SbMyb60*, a positive regulator of the lignin biosynthetic pathway in sorghum, resulted in the induction of many genes from the monolignol biosynthetic pathway, and led to increased lignin content and total energy [52, 53]. Herein, overexpression of *SbCCoAOMT* increased monolignol biosynthesis in sorghum, which led to changes in cell wall composition and an increase in the total energy of the biomass. These changes to cell wall composition could enhance sorghum biomass for a range of renewable fuel or chemical applications.

## Materials and methods

### Generation of transgenic *SbCCoAOMT* overexpression lines

The coding region of sorghum (*Sorghum bicolor*) *CCoAOMT* (Sobic010G052200.1) was amplified by PCR with the primers SbCCoAOMT_PciI-F, 5**ʹ**-CCGACATGTCCACCACGGCGACCGAG-3ʹ; and SbCCoAOMT_XbaI-R 5’- TGTTCTAGATCACTTGACGCGGCGGCA-3’, using Turbo *Pfu* polymerase (Agilent) and University of Georgia EST clone PH1_9_G09_A002 (GenBank accession CF428636) as the template. The coding region was subcloned between the E35S CaMV promoter and the 35S CaMV terminator as a *Pci*I and *Xba*I fragment and subjected to automated DNA sequencing to confirm DNA sequence fidelity. The pZP211 binary vector containing an *E35S*::*SbCCoAOMT* cassette was transformed into *Sorghum bicolor* (RTx430; grain; [54]) using *Agrobacterium tumefaciens* as described in Scully et al. (2016). Two independent transformation events (ZG 234-3-9A and ZG 234-1-28B), referred to as *SbCCoAOMT-*9a and *SbCCoAOMT-*28b, were selected for further characterization from eleven independent events based on robust *CCoAOMT* expression, CCoAOMT protein accumulation and identification on homozygous lines.

### Plant materials and growth conditions

Seeds (T3 generation) for each transgenic and wild-type (RTx430) lines were planted in a soil mixture with a 1:2:1:1 ratio of soil: peat moss: vermiculite: sand and arranged in a randomized complete block design at the University of Nebraska-Lincoln greenhouse facility. Plants were grown under a 16:8 h light:dark cycle and supplemented with high-pressure sodium lights. Greenhouse temperatures were maintained at 29–30°C and 26–27°C during day and night, respectively. Watering was conducted as needed and fertilization (Dyna Green All Purpose 12-12-12) was applied weekly. Two sets of plants were grown, one set of plants for an early harvest at 5 to 6-weeks and a second set grown to maturity. For the first set of sampled plants, the fifth leaf from the base and 10 cm of stalk tissue were harvested, immediately flash-frozen in liquid nitrogen, ground using a freezer mill (SPEX SamplePrep) and stored at -80°C for RNA-seq, Western blots and metabolomics. Additional leaf and stalk material were also collected from this group of plants for microscopy. The second set of plants were grown to maturity, seed heads were separated from stover biomass and all tissue was dried in forced-air ovens at 50°C. Experiments were conducted at the University of Nebraska Lincoln in Lincoln, Nebraska. Biomass was subsequently ground in a Wiley mill fitted with a 2-mm mesh screen (Arthur H. Thomas Co), followed by grinding on a cyclone mill fitted with a 1-mm mesh screen (UDY Co.) and stored for fiber, bomb calorimetry, thioacidolysis and phenolics analyses.

### RNA extraction, library preparation and sequencing

Total RNA was extracted from leaf and stalk tissue from three individual plants per line. Approximately 100 mg of homogenized plant material was added to 1 ml of TriPure Isolation Reagent (Roche Diagnostics) then RNA was extracted and purified using the RNA Clean and Concentrator Kit (Zymo Research). RNA was treated with an on-column DNase treatment (Zymo Research). RNA quality was assessed using an Agilent 2100 Bioanalyzer (Agilent Technologies) and two micrograms of total RNA per sample were utilized for TruSeq^™^ library preparation and RNA sequencing on an Illumina HiSeq2500 platform, generating 100 bp single-end reads. The barcoded libraries were multiplexed and sequenced across two lanes. RNA-Seq libraries, indexing and sequencing were performed at the University of Nebraska Medical Center DNA Sequencing Core Facility, Omaha, NE (https://www.unmc.edu/vcr/cores/vcr-cores/genomics/next-generation/index.html).

### Sequence analysis, assembly and differential expression analysis

High quality Illumina reads were mapped to the *S*. *bicolor* genome v3.1 (phytozome.jgi.doe.gov/pz/portal.html) using HISAT2 v2.0.5 (Kim et al., 2015) with default parameters (S1 Table). Files containing mapped reads were sorted and formatted for downstream analysis using SAMtools v1.3.1 (Li and Durbin, 2009) and the Subread v1.5.1, program *featureCounts* (Liao et al., 2013) was used to generate the count matrix for differential expression analysis. Differential expression analyses were performed using DESeq2 package v1.14.1 (Love et al., 2014) implemented in the R statistical environment v3.3.2 (R Development Core Team 2013). A principal components analysis was also used to depict the relationships between the *SbCCoAOMT* lines and wild-type and the variability among biological reps from the same treatment. Count data for statistical analysis were normalized using DESeq2 default settings and a variance stabilizing transformation was applied to correct for heteroscedasticity (Love et al., 2014).

Genes expressed at low-levels (less than 1 count across samples) were removed from the count matrix and differentially expressed genes between *SbCCoAOMT* and wild-type stalks and leaves were identified at a FDR adjusted *p*-value ≤0.05 using Wald tests (Love et al., 2014). Gene annotations from *S*. *bicolor* v3.1 genome were retrieved from Phytozome (phytozome.jgi.doe.gov/pz/portal.html) and matched to the expressed genes using R scripts. Weighted gene co-expression network analysis (WGCNA, version 1.43) [55] was used to identify groups of DEGS with similar expression patterns across the two *SbCCoAOMT* overpression lines and tissues as described in [53]. Briefly, the following parameters were used with the blockwiseModules function: TOMtype = “signed”, mergeCutHeight = 0.25, minModuleSize = 30. The RNA-seq datasets analyzed for this study are available at NCBI’s Sequence Read Archive under SRP158629.

### Histological staining and microscopy

Midribs from the fifth leaf and stalk tissue taken from ~10 cm from the bottom of each plant were collected from 5 to 6-week old transgenic and wild-type plants. Tissues were fixed in Ethanol: acetic acid (3:1 v/v), embedded in 7% agarose and 100 μM sections were cut using a Leica VT1200s vibratome (Leica Microsystems). Sections were hydrated for 30 min and stained for 15 s in phloroglucinol-20% HCl. Sections were imaged using an Olympus BX-51 light microscope (Olympus Co.) at 4x magnification. For confocal imaging, sections were mounted in water on glass slides and imaged with a Nikon A1R confocal laser scanning microscope (Nikon Instruments Inc.) using 405 and 488 nm lasers at 20x magnification to observe the autofluorescence. Identical settings were used for all samples.

### Western blot and immunodetection

Proteins from *SbCCoAOMT* overexpression lines and wild-type plants were isolated from ground leaf and stalk tissue collected from the first set of greenhouse grown plants. Proteins were extracted using an extraction buffer containing protease inhibitor (Sigma-Aldrich Co. P9599) (Sattler et al., 2009). Protein concentrations were measured using the Pierce 660nm Protein Assay (Thermo Fisher Scientific). Western blot analysis was conducted as previously described in Sattler et al. (2009). Briefly, the membrane was probed with primary antibody (polyclonal rabbit anti-SbCCoAOMT) at a 1:1000 dilution. Actin content was used as a loading control, and determined using a mouse anti-Actin monoclonal antibody (Sigma-Aldrich Co., A0480) at a 1:20,000 dilution. The secondary antibodies goat anti-rabbit (CCoAOMT; Sigma-Aldrich Co., A0545) and goat anti-mouse (Actin) IgG + horseradish peroxidase (Sigma-Aldrich Co., A4416) were used at dilutions of 1:8000 and 1:20,000, respectively. The secondary antibody was detected using chemiluminescence with Amersham ECL Western blotting reagent (GE Healthcare). Imaging of chemiluminescence was performed on a BioRAD ChemiDoc XRS+ instrument (BioRAD).

### Phenotypic evaluation and fiber analysis

Phenotypic traits were measured on the second set of plants grown to maturity. Day of inflorescence emergence was recorded, and height and numbers of tillers were measured immediately before harvest. Seeds were removed at maturity and total seed weight was measured. Total number of seeds was estimated for a given plant by weighing 100 seeds per plant and dividing total seed mass by mass for 100 seeds then multiplying by 100. Water and ethanol extractives, structural carbohydrates (cellulose, xylan, galactans, and arabinan), Klason lignin (e.g. acid insoluble lignin), and acetate were determined using the standard two-stage acid digestion protocol [56]. Moisture contents were determined by drying samples in a static oven at 105°C for 18–24 h. Sugars and acetate concentrations were measured using a Ultimate 3000 HPLC system equipped with a refractive index detector (Thermo Scientific, MA) an analytical column suitable for separation of sugars and organic acids (Aminex HPX-87H Column, 300 x 7.8 mm, Bio Rad Laboratories, Inc. Hercules, CA). Samples were injected at 20 μL and eluted with 5 mM sulfuric acid at 0.6 ml/min and 65°C. Fiber analysis was also performed on ground stover to determine cell wall components using a detergent digestion protocol as described by Vogel et al. (1999). Neutral detergent fiber (NDF), acid detergent fiber (ADF) and acid detergent lignin (ADL) concentrations were estimated using the ANKOM 200 fiber analyzer (ANKOM Tech Co.) (Vogel et al., 1999). Relative percentage of cell wall components were calculated using component concentrations extracted on a dry weight basis (Sarath et al., 2007). Stover from four biological replicates was analyzed in duplicate.

### Analysis by thioacidolysis

Stover from *SbCCoAOMT* transgenic and wild-type plants were treated for thioacidolysis followed by gas chromatography-mass spectrometry (GC-MS) to determine relative lignin subunit composition (*p-*hydroxyphenyl, guaiacyl, and syringyl lignin). Samples were prepared and analyzed as described in Palmer et al. (2008). Analysis was performed in duplicate on four biological replicates per line.

### Analysis of soluble and cell wall-bound phenolics

Soluble aromatic components were extracted from 100 mg of stover for transgenic and wild-type plants as described in Sarath et al. (2007). Briefly, soluble aromatic components were extracted using 1.5% acetic acid in 50% methanol. Wall-bound aromatics were extracted using residual plant material suspended in 4.0 M NaOH incubated at 90 °C for 2 h, released aromatics were extracted into ethyl acetate after acidification with 6.0 M HCl. Ethyl acetate extracts were vacuum-dried and each extract (soluble and wall-bound) were derivatized with trimethylsilyl (TMS) and trifluoroacetamide (MSTFA) (Thermo Fisher), and toluic acid was included in this reaction as an internal standard for quantification. The products were analyzed using GC-MS. Relative abundances of soluble and wall-bound phenolic compounds were determined by the peak areas of major ions. Between-sample normalization was performed using the peak area for the internal standard, toluic acid. Analysis was performed in duplicates on six biological replicates per line.

### Bomb calorimetry

Total energy content of stover was determined using a Parr 6400 bomb calorimeter (Parr Instrument Co.). Approximately 200 mg of dried plant material combined with 600 mg of mineral oil was combusted to estimate energy value per gram dry weight. Total energy of each biomass sample was calculated by subtracting the total energy released from combustion of mineral oil alone from the combined mineral oil and biomass sample and also standardizing by sample weight. Total energy was measured on six biological replicates in technical duplicates. In addition, to determine whether lignin or other cell wall components were contributing to energy differences between *SbCCoAOMT* overexpression lines and wild-type, energy levels were measured on stover from four biological replicates in technical duplicates after neutral detergent and acid detergent ANKOM washes.

### Metabolomics

Primary metabolites were extracted from 60–80 mg of leaf and stalk tissue from *35S*::*SbCCoAOMT* overexpression lines and wild-type plants with 80% MeOH in water. Samples were extracted by disruption of the ground tissues with 5–6 cycles with the Bullet Blender (Next Advance) by addition of 0.5 μm ZrO beads to each suspended sample. The extracts were centrifuged for 15 min at 15000 x *g* and 4°C and the supernatant was vialed and kept at 4 °C in the autosampler of an Agilent LC-1200 HPLC system (Agilent). LC-MS/MS data were acquired on a 4000QTrap (Sciex) operating in MRM mode. Waters Amide XBridge (4.6 x 100 mm, Milford, MA) was run at 0.5 mL min^-1^ with a linear gradient from 95% acetonitrile to 95% 20 mM Ammonium Acetate/Ammonium Hydroxide pH = 9.5 over 20 minutes. Analysis of samples in the positive and negative ionization modes were performed as separate injection sets and preprocessing was done for each ionization mode independently. Compounds identified in positive ionization mode were normalized with respect to ^15^N-labeled proline standard (2.31 μM) and are presented as nmol gram^-1^. Compounds identified in negative ionization mode were normalized with total ion chromatogram and are presented as % area gram^-1^. The list of putatively identified compounds (181 metabolites in both leaf and stalk) are listed in S2 Table.

### Statistical analysis

Statistical analysis of results from phenolics analysis, fiber analysis, bomb calorimetry, agronomic evaluations and thioacidolysis analysis were performed using “lmer” function of package “lme4” (Bates, 2005) in R (v3.3.2, R Foundation for Statistical Computing). Data were tested for normality using the Wilkes-Shapiro test in R and were log transformed if the data failed to meet normality. Pairwise comparisons among lines were performed using Tukey’s Honest Significant Differences test at α ≤0.05 using package “multicomp” [57].

## Results

### Overexpression of *SbCCoAOMT* in sorghum

**Fig 2:**
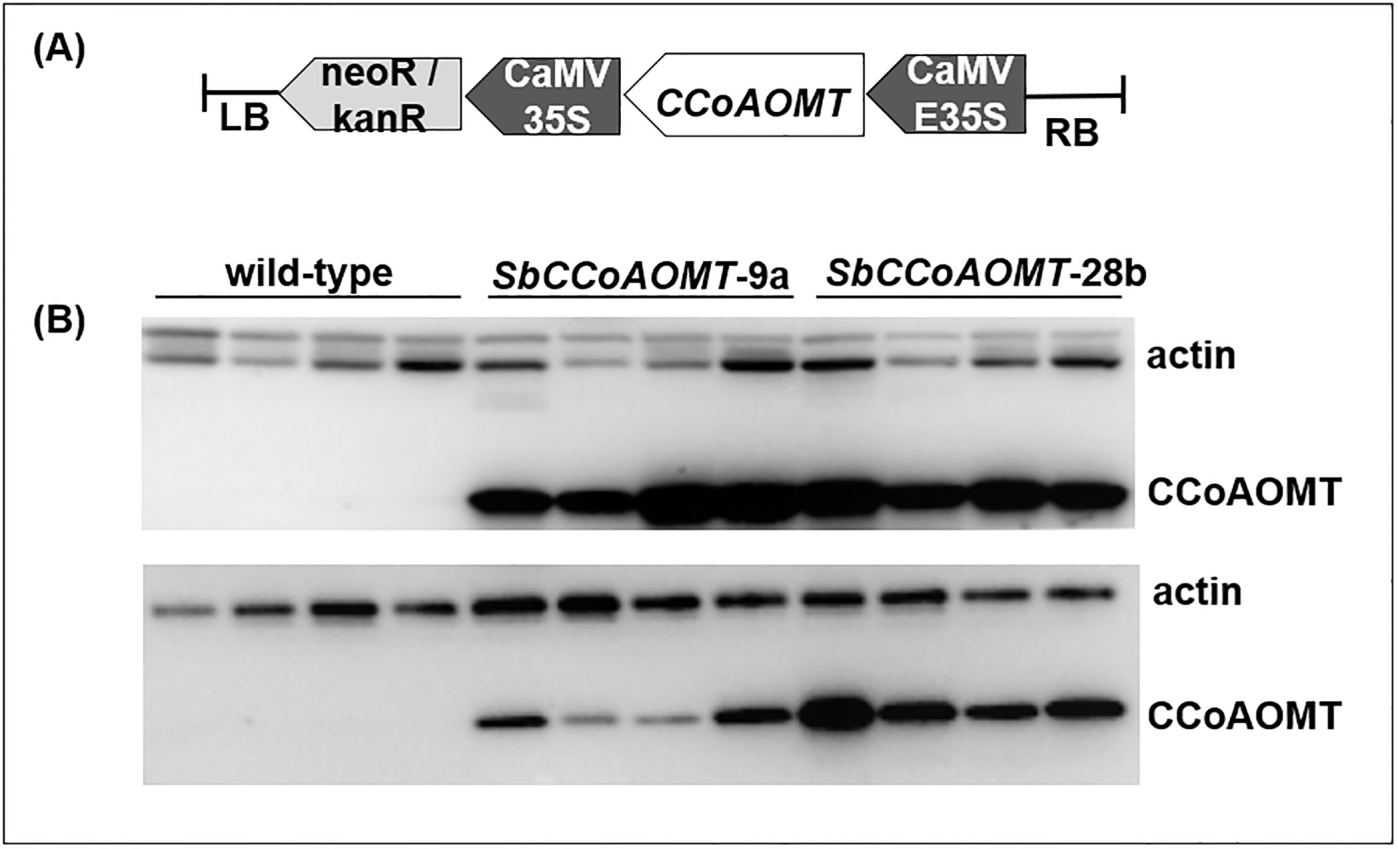
Overexpression of *SbCCoAOMT* in sorghum. (A) *35S*::*SbCCoAOMT* binary cassette used for sorghum (RTx430) transformation. T-DNA contained sorghum *SbCCoAOMT* gene (Sobic.010G052200.1) under the control of the E35S CAMV promoter with CAMV E35S terminator to end transcription. (B) Immunoblot detection of CCoAOMT from leaves (top) and stalks (bottom). Protein extracts from wild-type and *SbCCoAOMT* transgenic lines were separated by SDS-PAGE, transferred to membrane, and probed with polyclonal antibodies raised against the recombinant SbCCoAOMT protein. Each lane represents a biological replicate from homozygous lines. Monoclonal antibodies raised against actin protein were used as a protein loading control.

Previously structural and functional analysis of caffeoyl-CoA *O*-methyltransferase (SbCCoAOMT, Sobic.010G052200.1) indicated that this protein preferentially catalyzes *O*-methylation of hydroxy-cinnamoyl-CoA substrates, and is involved in monolignol biosynthesis in sorghum [30]. In order to determine the effects of increased *SbCCoAOMT* (Sobic.010G052200.1), *SbCCoAOMT* was overexpressed in one of the very few transformable sorghum lines, RTx430 under the control of constitutive E35S promoter (Fig 2A). Based on robust CCoAOMT protein accumulation two of the eleven transformant events, *SbCCoAOMT*-9a and *SbCCoAOMT*-28b, were further characterized. Immunoblot analysis was performed on protein accumulation in leaves and stalks using the polyclonal antibody against CCoAOMT (Fig 2B). Monoclonal antibodies against actin were used as a loading control, and the intensity of the actin bands was relatively consistent among all samples within each tissue type. The bands corresponding to the CCoAOMT protein were highly abundant in both the *SbCCoAOMT*-9a and *SbCCoAOMT*-28b (Fig 2B). In contrast, these bands were not visible in wild-type leaf and stalk extracts at this exposure interval, however, the protein was detected in wild-type leave and stalk extracts exposed for a longer time interval (S1 Fig). This experiment indicated that overexpression of *SbCCoAOMT* resulted in an increased accumulation of this protein in both leaf and stalk tissue for both of the events evaluated.

### Phenotypic and stover analysis of *SbCCoAOMT* overexpression lines

**Fig 3:**
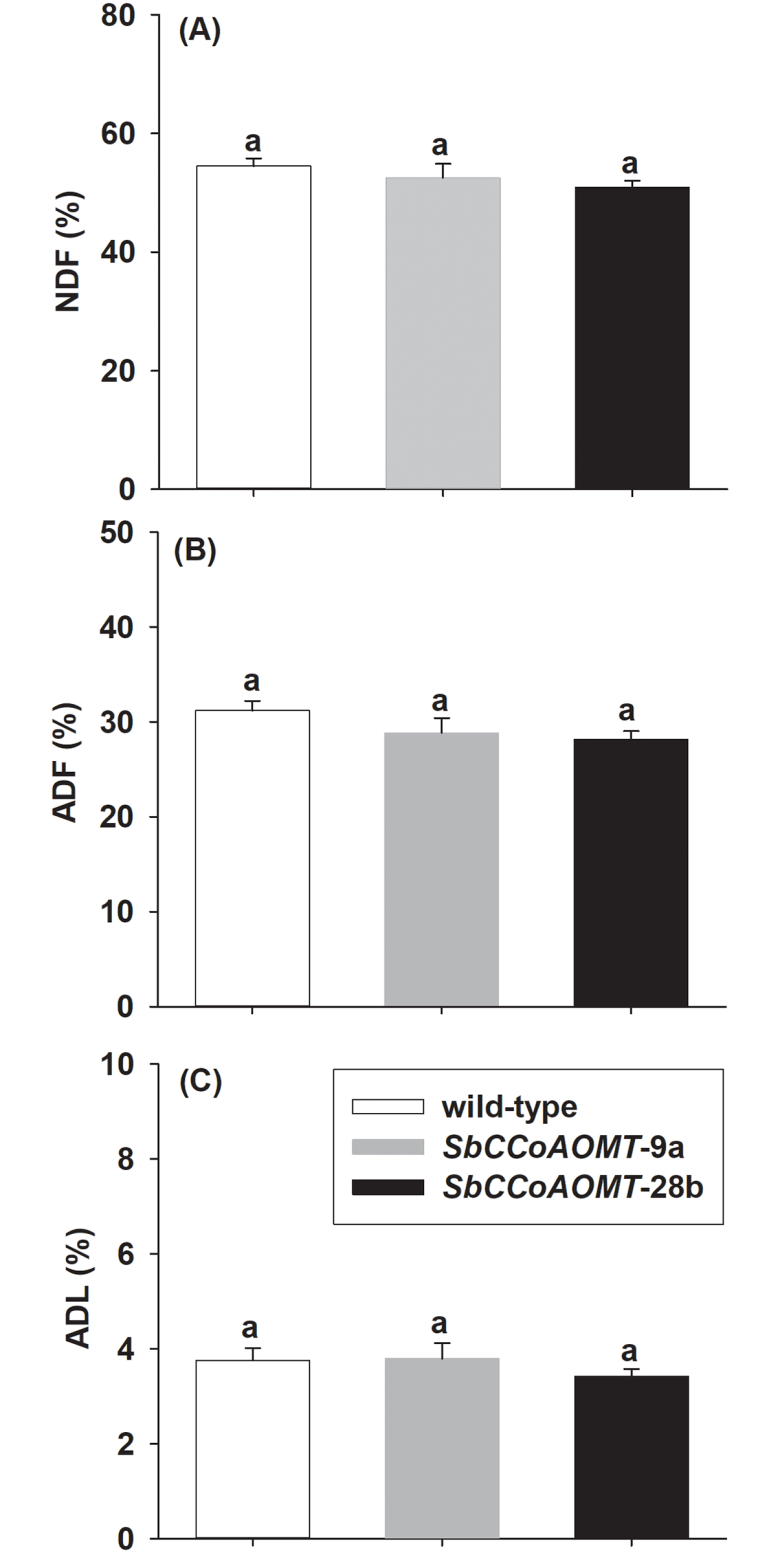
Analysis of mature stover from wild-type and *35S*::*SbCCoAOMT* transgenic plants (A) neutral detergent fiber (NDF), (B) acid detergent fiber (ADF) and (C) acid detergent lignin (ADL). NDF, ADF and ADL determined using an ANKOM fiber analyzer. Values presented are least square means (+1 SE). Samples with different letters are statistically different from one another at α ≤ 0.05 using Tukey’s HSD test.

We observed no significant changes in growth and development associated with overexpression of *SbCCoAOMT* when comparing either transgenic line with the wild-type (Table 1). We also found overexpression of *SbCCoAOMT* did not trigger significant variation in monomer composition of lignin with similar levels of H-, G- and S-lignin subunits and S:G ratio across wild-type and transgenic lines (Table 2). Similarly, the lignin concentration did not differ in the *SbCCoAOMT* overexpression lines relative to wild-type, which was determined by the two methods Klason lignin (Acid Insoluble Lignin) (*p* = 0.8268) (Table 3) and acid detergent lignin (ADL) (*p* = 0.5564) (Fig 3C), and was consistent with the lack of differences in lignin monomer concentrations. Overexpression of *SbCCoAOMT* also did not affect the abundances of structural carbohydrates in stover (Table 3). Likewise, fiber analysis revealed levels of NDF and ADF were not significantly different between *SbCCoAOMT* and wild-type (*p* = 0.4066 and *p* = 0.2576, respectively) (Fig 3A and 3B).

**Table 1:** Agronomic traits of wild-type (RTx430) and *35S*::*SbCCoAOMT* plants.

	Wild-type (± 1 SE)	*SbCCoAOMT*-9a (± 1 SE)	*SbCCoAOMT*-28b (± 1 SE)	*p*-value
Inflorescence emergence (days)	110.63 (1.04)	115.75 (1.56)	113.50 (1.56)	0.0613
Plant height (cm)	102.13 (3.46)	94.08 (4.66)	92.08 (4.66)	0.1230
Number of tillers	2.00 (0.29)	2.00 (0.43)	1.75 (0.43)	0.8761
Total seed weight (g)	50.71 (4.91)	46.34 (7.32)	59.14 (7.32)	0.4461
Estimated total number of seeds	1244.40 (123.09)	1174.78 (183.49)	1318.79 (183.49)	0.8458

Values presented represent least square means (lsmean) (± 1 SE).

**Table 2:** Lignin composition determined via thioacidolysis GC-MS for wild-type (RTx430) and *35S*::*SbCCoAOMT* stover.

	Wild-type (± 1SE)	*SbCCoAOMT*-9a (± 1SE)	*SbCCoAOMT*-28b (± 1SE)	*p-*value
H-lignin	0.045 (0.014)	0.045 (0.006)	0.050 (0.008)	0.8514
G-lignin	1.428 (0.378)	1.519 (0.271)	1.659 (0.271)	0.8350
S-lignin	0.949 (0.331)	1.048 (0.185)	0.971 (0.154)	0.9378
S/G ratio	0.618 (0.063)	0.715 (0.041)	0.587 (0.016)	0.1420

The relative abundance of peak area ion for *p-*hydroxyphenyl (H-lignin), guaiacyl (G-lignin) and syringyl (S-lignin) subunits to internal standard (4,4’-Ethylidenebisphenol) determined by GC/MS. Values presented represent least square means (lsmean) (± 1 SE).

**Table 3:** Plant cell wall traits of wild-type (RTx430) and 35S::SbCCoAOMT plants.

Component (mg g^-1^)	Wild-type (± 1SE)	*SbCCoAOMT*-9a (± 1SE)	*SbCCoAOMT*-28b (± 1SE)	*p-*value
Extractables	310.92 (10.87)	401.45 (124.55)	400.86 (15.54)	0.8522
Glucan	236.36 (5.44)	227.85 (15.43)	224.41 (6.10)	0.6990
Xylan	139.69 (4.53)	136.01 (10.10)	132.89 (3.41)	0.7766
Arabinan	13.95 (1.56)	14.64 (1.56)	14.01 (0.91)	0.9263
Galactan	5.34 (0.29)	5.59 (0.32)	6.51 (0.29)	0.0492
Acetate	5.82 (1.23)	12.47 (4.60)	13.44 (4.64)	0.3544
Acid Soluble Lignin	8.69 (0.25)	8.82 (0.56)	9.38 (0.21)	0.4188
Acid Insoluble Lignin	91.15 (1.50)	88.90 (3.59)	88.89 (3.31)	0.8268
Sum (out of 1000)	917.21 (11.52)	923.86 (11.20)	915.84 (7.67)	

The abundances of cell wall components were measured according to the analytical procedure of the National Renewable Energy Laboratory. All values are represented in terms of mg g^-1^ of biomass. Values presented represent least square means (lsmean) (± 1 SE).

**Fig 4:**
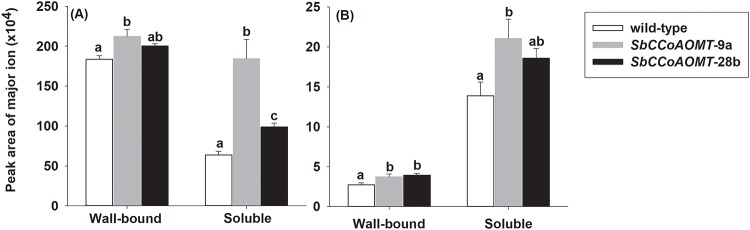
Relative abundance of wall-bound and soluble (A) ferulic acid and (B) sinapic acid. Ferulic acid and sinapic acid was analyzed via GC-MS. Values presented are least square means (+ 1 SE). Samples with different letters for wall-bound and soluble fractions are statistically different from one another at α ≤ 0.05 using Tukey’s HSD test.

To better characterize changes made to the phenolic components of cell walls, soluble and cell wall-bound phenolic compounds were extracted from mature stover and relative abundance of several phenolic compounds including compounds derived from the monolignol pathway and several other organic acids was measured by GC-MS (S3 Table). While lignin content and composition did not differ between overexpression lines and wild-type plants, phenolic compounds were clearly different with respect to wall-bound and soluble residues. Notably, the levels of cell wall-bound and soluble ferulic acid were significantly higher (*p* = 0.0248 and *p ≤* 0.001, respectively) between transgenic lines and wild-type (Fig 4A), soluble ferulic acid levels in *SbCCoAOMT-*9a and *SbCCoAOMT-*28b stover were 2.0 and 0.5-fold higherthan wild-type, respectively (Fig 4A). Sinapic acid was also significantly greater in *SbCCoAOMT-*9a and *SbCCoAOMT-*28b stover than wild-type for both cell wall-bound and soluble fractions (*p* = 0.0361 and *p* = 0.0442, respectively; Fig 4B). Soluble sinapic acid relative to wild-type were 0.6 and 0.4- fold higher in *SbCCoAOMT-*9a and *SbCCoAOMT-*28b, respectively (Fig 4B), while wall bound levels of sinapic acid were 0.4-fold higher in both lines relative to wild-type. One other soluble phenolic, *p-*hydroxymandelic acid, was found to be significantly higher in *SbCCoAOMT*-28b than wild-type with a 50% increase (S3 Table). The phenolic composition suggests that overexpression of *SbCCoAOMT* increased levels of two phenolic acids from the monolignol biosynthesis pathway, which were incorporated into the cell wall.

**Fig 5:**
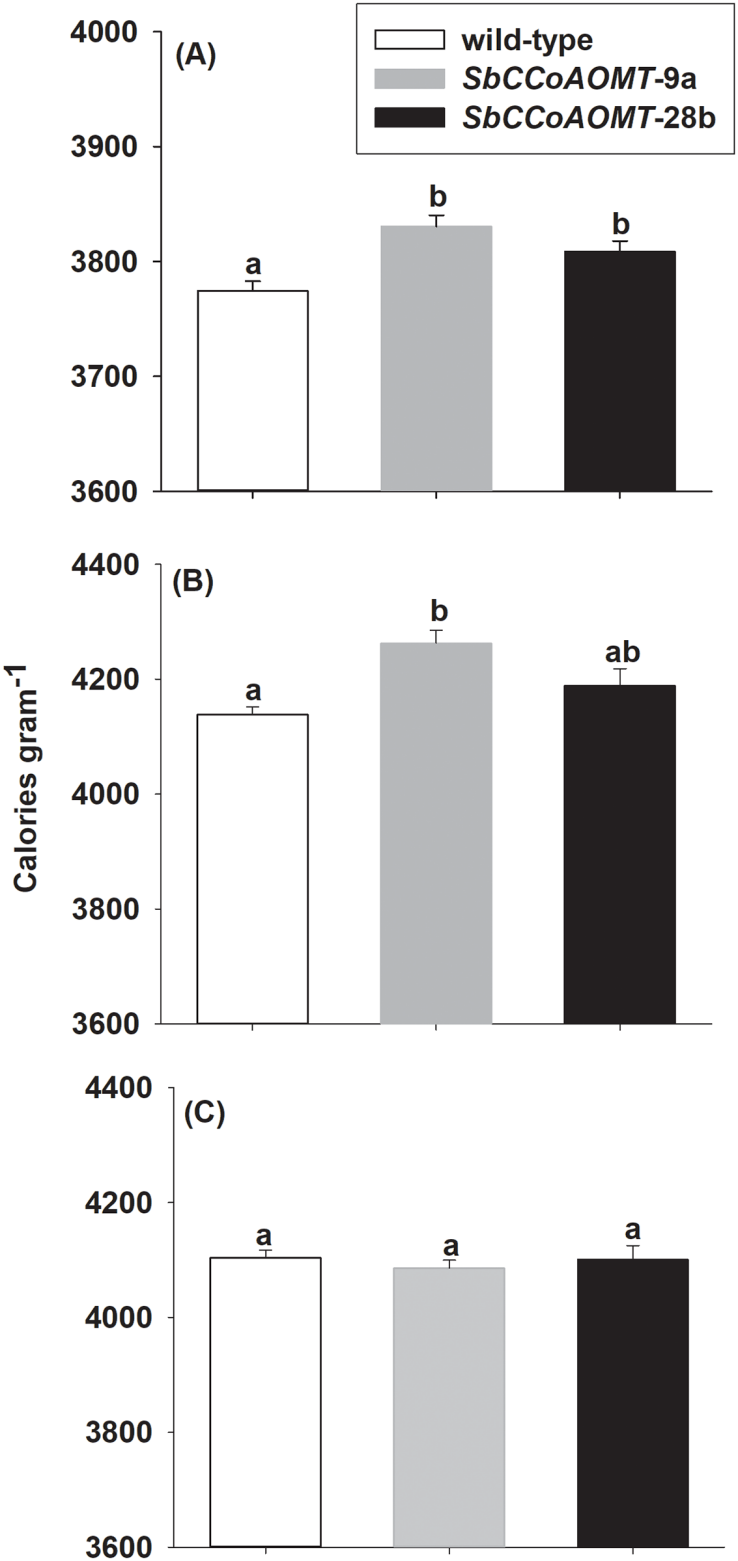
Total energy from wild-type and *35S*::*SbCCoAOMT* (A) mature stover, (B) after neutral detergent fiber wash and (C) after neutral detergent and acid detergent washes. Total energy content was determined using a Parr 6400 bomb calorimeter. Values presented are least square means (+ 1 SE). Samples with different letters are statistically different from one another at α ≤ 0.05 using Tukey’s HSD test.

Total energy of stover harvested from *SbCCoAOMT* overexpression lines was 61 and 75 cal g^-1^ greater than wild-type for *SbCCoAOMT-*28b and *SbCCoAOMT-*9a, respectively (*p* = 0.006) (Fig 5A). To better characterize the source for the observed increase in total energy among the cells walls of the overexpression lines, total energy was measured from stover following NDF and ADF washes (Fig 5B and 5C). During the NDF wash, sugars, lipids, pectins, starches, soluble proteins and phenolics are removed (soluble components), leaving three cell wall polymers and wall-bound phenolics. Total energy concentrations for all lines increased after NDF, 9.4%, 10.3% and 9.1% for wild-type, *SbCCoAOMT-*9a and *SbCCoAOMT*-28b, respectively (Fig 5B). Total energy for *SbCCoAOMT-*9a NDF washed stover was greater than wild-type samples by 124 cal g^-1^ (*p* = 0.0094), but not significantly different between *SbCCoAOMT*-28b and wild-type NDF washed stover. By contrast after the ADF wash, which removes hemicellulose, wall-bound proteins and phenolic groups, total energy decreased by 0.85%, 4.3% and 2.2% for wild-type, *SbCCoAOMT-*9a and *SbCCoAOMT*-28b, respectively and did not differ between *SbCCoAOMT* transgenic lines and wild-type (*p* = 0.7668) (Fig 5C). This result indicated that the increased energy observed in the stover from *SbCCoAOMT-*9a overexpression line was derived from acid labile cell wall-bound moieties. Hence, the source for increased energy levels (Fig 5A and 5B) likely originates from the higher levels of wall-bound (esterified) phenolic acids (Fig 4A and 4B).

**Fig 6:**
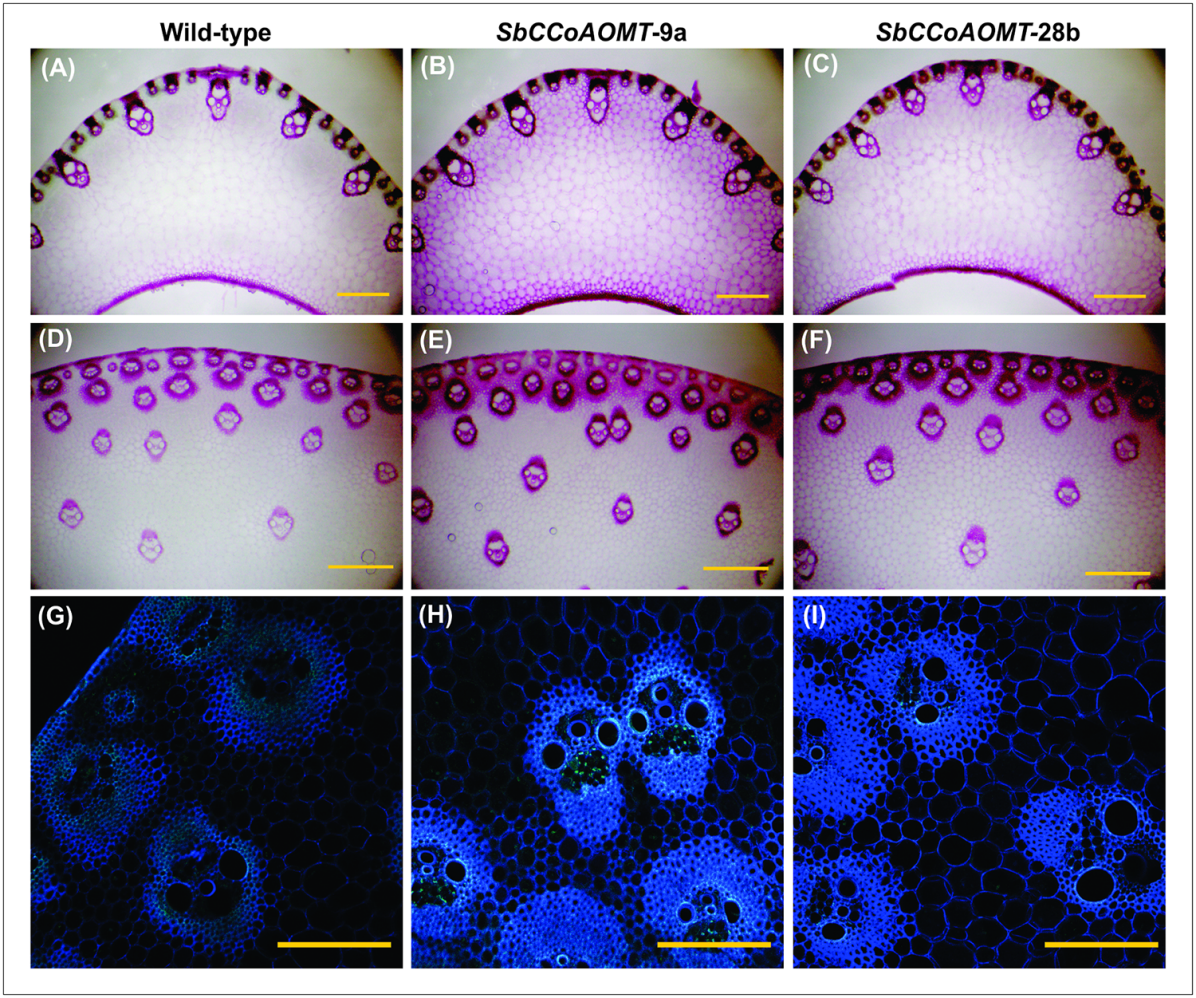
*SbCCoAOMT* overexpression induced changes in cell wall composition in leaves and stalk tissue. A to F, visualization of cell wall after phloroglucinol-staining of leaf midrib (A-C) and stalk tissue (D-F) taken from wild-type and *35S*::*SbCCoAOMT* transgenic plants (indicated on top of each panel). G to I, autofluorescence observed with a Nikon A1R confocal laser scanning microscope from wild-type and *35S*::*SbCCoAOMT* stalk cross-sections. Scale bar = 500 μm (A-F); 200 μm (G-I).

The cell walls from leaf midrib and stalk cross-sections were observed using phloroglucinol staining to visualize lignin and phenolic groups. Both *SbCCoAOMT* overexpression lines showed more intense staining than wild-type. Increased phloroglucinol staining was observed around the vascular bundles of both overexpression lines compared to wild-type in both the leaf midrib (Fig 6A–6C) and stalk (Fig 6D–6F). The parenchyma cells of the leaf midrib sections also showed greater phloroglucinol staining in sections from *35S*::*SbCCoAOMT* lines relative to wild-type, which was especially evident in the cross sections from *SbCCoAOMT-*9a plants (Fig 6B). The increased staining is likely due to phloroglucinol reactive groups other than lignin within the cell walls of the *SbCCoAOMT* overexpression lines, because lignin (ADL and Klason lignin) did not differ between *SbCCoAOMT* and wild-type (Fig 3C and Table 3). Confocal microscopy was used to visualize cell wall associated phenolic compounds through autofluorescence, and many compounds of monolignol biosynthesis fluoresce when illuminated by ultraviolet light [58, 59]. Developing stalk cross-sections in both *SbCCoAOMT*-9a and *SbCCoAOMT-*28b showed greater autofluoresence in vascular bundles and to a lesser extent the parenchyma cells surrounding the vascular bundles compared to wild-type (Fig 6G–6I). These differences were more pronounced in the *SbCCoAOMT*-9a line compared to wild-type. No significant alterations in levels of autofluorescence were observed from leaf midrib sections between wild-type and both transgenic events (S2 Fig). Together, the phloroglucinol staining and autofluorescence indicated *SbCCoAOMT* overexpression increased the deposition of phenolic compounds within sorghum cell walls.

### Effect of *SbCCoAOMT* overexpression on monolignol biosynthetic genes, transcriptome and metabolome

**Fig 7:**
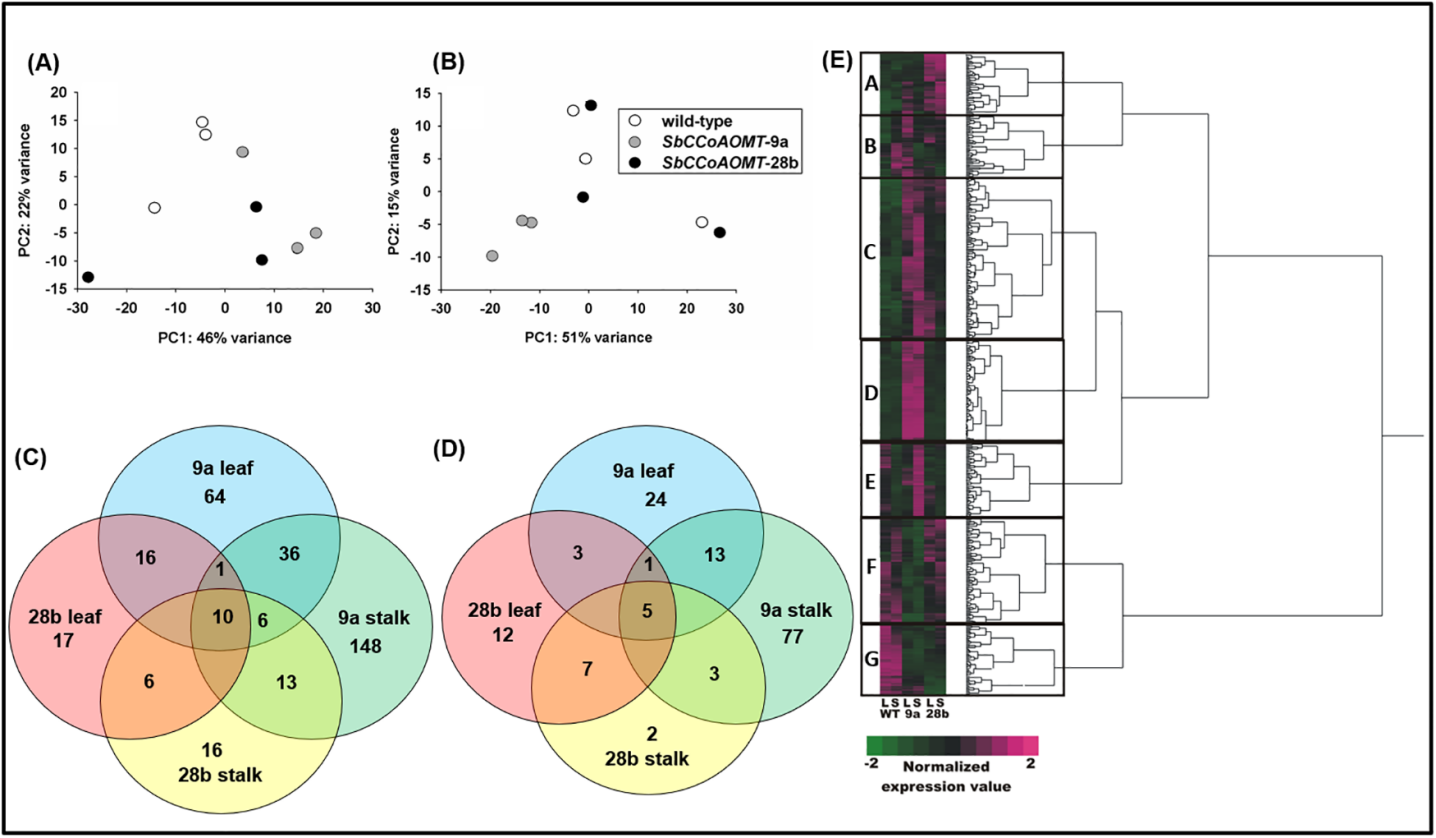
Overview of differentially expressed genes for *SbCCoAOMT-*9a (9a) and *SbCCoAOMT*-28b (28b) in leaf and stalk tissue compared to wild-type. Principal components analysis of RNA-seq data on individual samples from wild-type and *SbCCoAOMT* in (A) leaf and (B) stalk tissue. Venn diagrams of (C) increased and (D) decreased genes detected in RNA-seq experiments. (E) Heatmap analysis of differentially expressed genes in leaves (L) and stalks (S) for wild-type (WT), *SbCCoAOMT*-9a (9a) and *SbCCoAOMT*-28b (28b). Differentially expressed genes were determined using DESeq2 with a threshold of FDR <0.05; LFC > 1.0. Numbers within regions in venn diagram indicate common and unique genes within each sector. Raw counts of genes that were differentially expressed in at least one *35*::*SbCCoAOMT* line relative to wild-type were log-transformed and Z-score standardized for a normalized expression value. Heatmap was prepared using hierarchial clustering analysis in JMP 12.2.0 (SAS Institute Inc.). Letters (A-G) within heatmap indicate hierarchial clusters of genes.

Overall, there were no major differences in the number of mapped RNA-seq reads between tissues or among the three lines (S1 Table). The transcriptomes of *35S*::*SbCCoAOMT* transgenic leaves were partially differentiated from wild-type plants along the second principal component (PC2, which accounted for 22% of the variance) (Fig 7A). In stalk, *SbCCoAOMT*-9a could be somewhat differentiated from wild-type while the stalk transcriptomes of *SbCCoAOMT*-28b and wild-type overlapped significantly (PC1, which accounted for 51% of the variance) (Fig 7B). This result indicated that overexpression of *SbCCoAOMT* did not significantly impact the global expression patterns relative to wild-type. In corroboration with the RNA-seq principal component analysis there were a total of 480 genes differentially expressed among either *SbCCoAOMT* lines compared to wild-type with 333 genes up-regulated and 147 genes down-regulated (Fig 7C–7E and S4 Table). *SbCCoAOMT* overexpression increased and decreased expression of 1.0% and 0.4% of all genes, respectively based upon ~34,496 genes annotated in the sorghum genome [2]. 133 and 214 genes were identified as up-regulated in *SbCCoAOMT*-9a leaves and stalk tissue, respectively compared to wild-type and 46 and 99 genes down-regulated in *SbCCoAOMT*-9a leaves and stalk tissue, respectively compared to wild-type (Fig 7C–7E and S4 Table). *SbCCoAOMT*-28b leaves and stalk tissue had fewer differentially expressed genes compared to wild-type with 50 and 51 genes up-regulated and 28 and 17 genes down-regulated in leaves and stalk tissue, respectively. Only 15 genes of the 480 differentially expressed genes were impacted in both transgenic events and both tissue types (Fig 7C and 7D; S4 Table). In *SbCCoAOMT*-9a and *SbCCoAOMT*-28b leaves, 221 genes were differentially expressed in leaf tissue from both lines while 344 genes were differentially expressed in stalk tissue from both events. Hierarchical clustering analysis of the 480 DEGs could be broadly segregated into 7 distinct clusters based on expression patterns across tissue type (labeled A-G; Fig 7E). Most clusters showed patterns of up-regulated or down-regulated genes that were specific to a single line, however were not always consistent across *SbCCoAOMT* overexpression lines. Clusters C and G were the exception, which contained genes with expression patterns consistent across both *SbCCoAOMT* overexpression lines. Genes encoding for acyl-CoA N-acyltransferase (Sobic.005G055150), 3-deoxy-o-arabino-heptulosonate phosphate synthase (DAHP; Sobic.001G351000), a peroxidase precursor (Sobic.001G235800), several glycosyl transferases (Sobic.001G083900, Sobic.003G297700 and Sobic.004G224400) and shikimate kinase (Sobic.006G235500) were contained in cluster C and elevated in both *35S*::*SbCCoAOMT* lines compared to wild-type plants (S4 Table). Acyl-CoA N-acyltransferase transcript levels were elevated in leaves and stalk tissue for *SbCCoAOMT-*9a and *SbCCoAOMT-*28b relative to wild-type. The expression of DAHP synthase and shikimate kinase genes, which is involved in aromatic amino acid synthesis, were increased only in *SbCCoAOMT-*9a and *SbCCoAOMT-*28b stalks with relative to wild-type. Transcripts of a peroxidase precursor (Sobic.001G235800), assigned to phenylpropanoid biosynthesis KEGG pathway, were elevated in stalk tissue relative to wild-type for both *SbCCoAOMT-*9a and *SbCCoAOMT-*28b. Cluster G contained 50 genes that are down-regulated in *SbCCoAOMT* overexpression lines relative to wild-type plants, and based on KEGG pathway there are single genes involved with endocytosis (K07904; Sobic.009G003800), plant-pathogen interaction (K13447; Sobic.007G148300) and ubiquitin mediated proteolysis (K04506; Sobic.003G092500).

**Fig 8:**
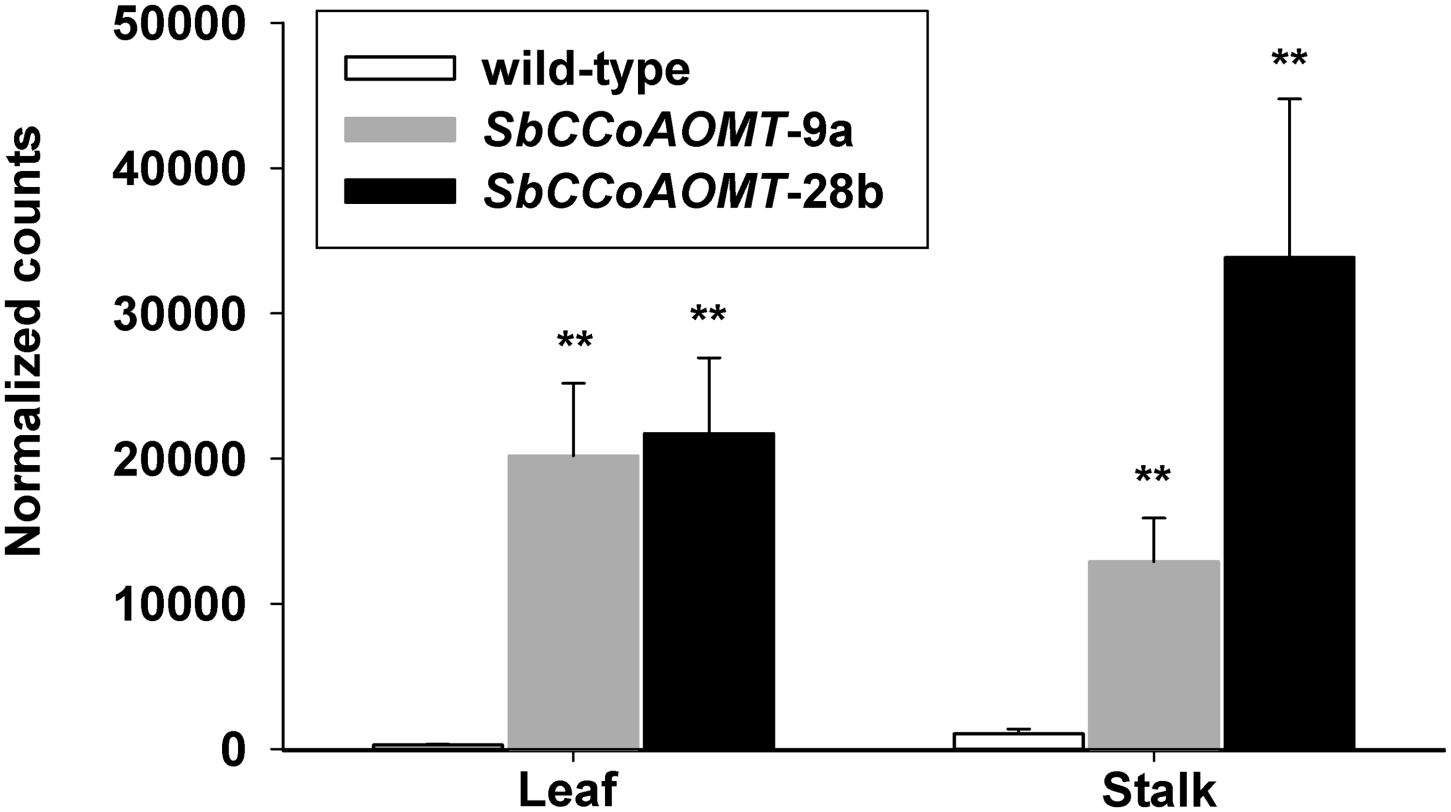
Impact of *SbCCoAOMT* overexpression on *SbCCoAOMT*. Expression of caffeoyl-coA *O-*methyltransferase (*CCoAOMT*; Sobic.010G052200.1) were quantified using the RNA-seq dataset. Asterisks indicate levels of significance for differential expression of *SbCCoAOMT* transgenic event compared to wild-type determined using DESeq2 (FDR: ** *p* ≤ 0.01).

Even though overexpression of *SbCCoAOMT* did not appear to significantly modulate the global expression patterns, the expression profiles of the major ten genes involved in the monolignol biosynthetic pathway were evaluated. These genes are identified as the major genes in the monolignol biosynthetic pathway based on amino acid similarity of previously identified genes and on robust gene expression in stalk tissue [52, 60]. *CCoAOMT* transcript levels in leaves showed an approximately 60-fold increase relative to wild-type for *SbCCoAOMT-*9a and *SbCCoAOMT-*28b with no significant differences between the two lines (Fig 8). However, in stalks, there were more moderate increases in *CCoAOMT* transcript levels that were 12.5 and 33-fold higher in *SbCCoAOMT-*9a and *SbCCoAOMT-*28b relative to wild-type. In stalks, mean expression levels of *CCoAOMT* in *SbCCoAOMT*-28b were 89.7% greater than *SbCCoAOMT-*9a (Fig 8). Although *SbCCoAOMT* expression was consistently higher for both transgenic events in both tissues, *SbCCoAOMT* expression in stalk tissue exhibited greater variability relative to leaf. Phenylalanine ammonia lyase (PAL) is the first enzyme of the monolignol biosynthetic pathway that catalyzes the deamination of phenylalanine to trans-cinnamic acid, a precursor for the lignin, flavonoid and other phenylpropanoid biosynthetic pathways. Expression levels of the most highly expressed PAL gene (Sobic.004G220300) in both stalk and leaf tissue were significantly greater in the *SbCCoAOMT-*9a transgenic line than wild-type (S3 Fig). Other PAL-like genes (Sobic.004G220600, Sobic.005G134501, Sobic.004G220700 and Sobic.004G220400) also had greater transcript levels in *SbCCoAOMT*-9a leaf and stalk tissues (S4 Table). Besides PAL, the expression levels of other genes in the monolignol biosynthesis pathway did not differ between *35S*::*SbCCoAOMT* and wild-type plants in either stalk or leaf tissues (S3 Fig).

**Fig 9:**
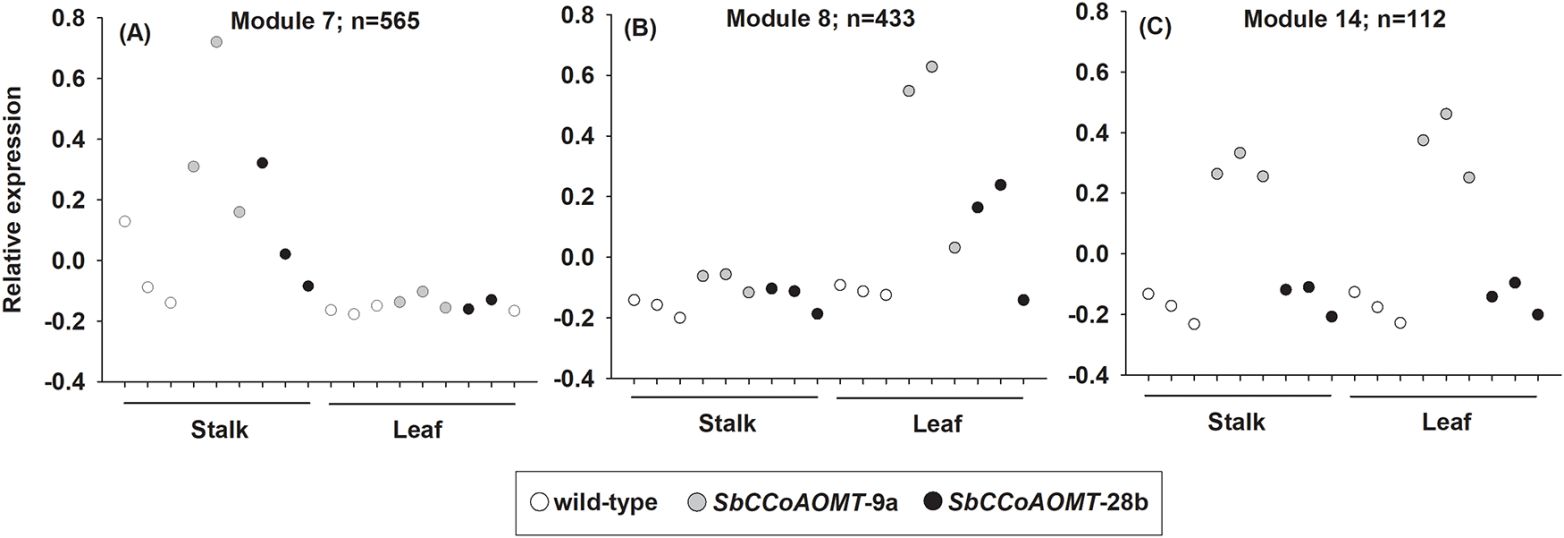
Weighted gene co-expression network analysis (WGCNA) of differentially expressed genes in sorghum *35S*::*SbCCoAOMT* stalks and leaves. Expression patterns of genes assigned to co-expression module (A) 7, (B) 8 and (C) 14. Module 7 and 8 represent genes upregulated in *SbCCoAOMT* stalk and leaves, respectively, whereas module 14 are upregulated in *SbCCoAOMT*-9a stalks and leaves.

Weighted gene co-expression network analysis (WGNCA) further illustrated the minor impacts of *SbCCoAOMT* overexpression on sorghum stalks and leaves. Three out of a total of 23 co-expression modules identified were associated with *SbCCoAOMT* overexpression lines (Fig 9 and S4 Fig), which was divided into three primary groupings. Module 7 consisted of 565 genes that were up-regulated in *SbCCoAOMT* stalks relative to wild-type (Fig 9A), whereas module 8 consisted of 433 genes up-regulated in *SbCCoAOMT* leaves relative to wild-type (Fig 9B). Module 7 genes were predominantly related to pentose phosphate pathway, phenylalanine, tyrosine and tryptophan biosynthesis, starch and sucrose metabolism and phenylpropanoid biosynthesis KEGG pathways. Module 8 genes tended toward up-regulation in leaves of both *SbCCoAOMT* overexpression lines and were part of glycolysis, photosynthesis, purine and pyrimidine metabolism, starch and sucrose metabolism and porphyrin and chlorophyll metabolism KEGG pathways. Module 14 included 112 genes that are induced in *SbCCoAOMT*-9a leaves relative to wild-type (Fig 9C) and genes within this module were part of fatty acid elongation, phenylalanine metabolism and phenylpropanoid biosynthesis KEGG pathways. Although overexpression of *SbCCoAOMT* minimally impacted global gene expression, WCGNA identified expression patterns of phenylpropanoid-related gene that were increased in the overexpression lines.

The monolignol biosynthesis pathway requires phenylalanine or tyrosine as substrates and NADPH, SAM and CoA as cofactors; the metabolites associated with these pathways were further investigated. Phenylalanine is derived from the shikimate biosynthesis pathway, some of the metabolites involved in this pathway were altered. Concentrations of shikimate were significantly higher in stalk tissues from *SbCCoAOMT*-9a transgenic plants (S2 Table). Concentrations for other shikimate pathway metabolites detected via LC-MS included tryptophan, shikimate-3-phosphate, phenylalanine and phenyllactic acid, were not significantly different between overexpression lines and wild-type plants. Metabolites associated with NAD^+^ and NADP^+^ including aspartate, quinolinate, pyrophosphate, nicotinate, and glutamine were not significantly different between overexpression lines and wild-type plants. However, dihydroxy-acetone-phosphate was decreased in overexpression lines relative to wild-type leaf tissue and glutamate was increased in overexpression lines relative to wild-type stalk tissue. Concentrations of metabolites associated with *S*-adenosyl methionine (SAM) biosynthesis pathway 2-oxo-4-methylthiobutanoate, *S-*adenosyl-methioninamine and *S*-methyl-5-thioadenosine were not significantly different between overexpression lines and wild-type plants. Metabolites erythrose-4-phosphate and glucose-6-phosphate involved in the pentose phosphate pathway were not significantly different in *SbCCoAOMT* transgenic lines relative to wild-type in both leaves and stalk tissues, however, phosphoenolpyruvate, was significantly lower in *SbCCoAOMT-*28b leaves compared to wild-type plants. Metabolites associated with CoA metabolism were not significantly different between *SbCCoAOMT* transgenic lines and wild-type plants. In leaf tissue from *SbCCoAOMT* transgenic plants there are significant decreases in aconitate metabolites, which is an intermediate product of citrate to isocitrate and the basic way of citrate synthesis is with oxaloacetate condensation with acetyl-CoA. In general, less variability among the metabolite profiles was observed between biological replicates of leaves (S5A and S5B Fig) relative to stalks (S5C and S5D Fig).

## Discussion

The effects of *SbCCoAOMT* overexpression (Sobic.010G052200.1), whose gene product is a central enzyme of monolignol biosynthesis [61], were investigated in sorghum, an emerging C_4_ bioenergy crop. The results of this study demonstrated that overexpression of *SbCCoAOMT* was sufficient to stimulate the biosynthesis of ferulate and sinapate, which led to their incorporation into cell walls as soluble and esterified groups and increased energy content of sorghum stover.

Genes of the monolignol biosynthetic pathway have been targeted using antisense and RNAi engineering techniques to lower lignin content and alter lignin composition of plants and improve deconstruction of cell walls for the conversion into liquid biofuels [6, 62]. In contrast, few studies have evaluated the consequences of overexpression or ectopic expression of these genes in herbaceous plant species. This strategy provides a means to increase enzyme concentration and rate of reaction *in vivo*, which can lead to increased energy content of biomass for thermal conversion, and higher levels of phenolic compounds in biomass for chemical end-uses. Previously, the consequences of inducing lignin synthesis through the overexpression of a transcriptional activator *SbMyb60* were evaluated in grain sorghum. Overexpression of this transcription factor in sorghum resulted in increased lignin content and increased levels of aromatic compounds, which elevated total energy levels in sorghum biomass. However, overexpression of *SbMyb60* affected growth and development of sorghum resulting in reduced height, delayed flowering and altered plant architecture. [52, 53].

The overexpression of *CCoAOMT* overexpression in sorghum did not significantly affect growth, unlike the downregulation of *CCoAOMT* through RNAi or antisense tools other plant species [28, 50, 63]. Although this enzyme is required for the synthesis of both G and S subunits, *CCoAOMT* suppression in maize, alfalfa, Arabidopsis and tobacco led to reductions in G subunits, lignin concentrations and plant growth [28, 50, 51, 63]. Overall, levels of S lignin were not impacted in any of these studies. In contrast, our findings showed that stover from mature plants did not differ in terms of S/G ratio or levels of subunits S-lignin and G-lignin between *35S*::*SbCCoAOMT* and wild-type, which indicated the increased levels of *SbCCoAOMT* did not impact lignin composition. In addition, analysis of both Klason and ADL lignin concentrations demonstrated that lignin was unaffected in stover and no detectable differences in growth or development were observed in *SbCCoAOMT* overexpression lines. Thus, *CCoAOMT* overexpression alone is not sufficient to increase lignin or alter its composition in sorghum.

While overexpression of *SbCCoAOMT* did not impact lignin, it did increase the accumulation of soluble and wall-bound hydroxycinnamic acids in the stover. Both transgenic events accumulated higher levels of ferulate and sinapate in both cell wall-bound and soluble fractions from stover, which are products derived from monolignol biosynthesis. The higher levels of these compounds likely result from the accumulation of feruloyl-CoA, the product of CCoAOMT. Similarly, both ferulate and sinapate levels were elevated when *SbMyb60* was overexpressed *in planta* [52, 53]. Although the likely route from feruloyl-CoA to sinapic acid is through the monolignol biosynthesis enzymes CCR, F5H and COMT, alternative paths may exist. In alfalfa (*Medicago sativa*), MsCCoAOMT is able to methylate 5-hydroxyferuloyl-CoA [64], which could in part explain the increased levels of sinapic acid in the *CCoAOMT* overexpression lines. However, no enzymatic activity was detected with 5-hydroxyferuloyl-CoA as the substrate for SbCCoAOMT, and its protein structure and substrate docking indicated there are steric clashes within active site that likely prevent substrate binding [30]. Higher levels of esterified phenolic acids in the cell wall were also observed under fluorescence microscopy in the stalks from both transgenic events. Cell wall autofluorescence has been associated with esterified ferulic acid residues [59]. Thus, the increased autofluorescence and phloroglucinol staining observed in *SbCCoAOMT* transgenic stalks are likely the result of increased esterified ferulic acid and sinapic acid residues in cell walls. Deposition of esterified ferulate is also a plant defense response against biotic incursion [65], and the induction and accumulation of CCoAOMT transcripts and protein has previously been observed in pathogen incursion [66, 67]. Here, *SbCCoAOMT* overexpression resulted in the accumulation of cell wall-bound ferulic acid without an elicited defense response, which could ultimately lead to increased resistance to pests and pathogens.

An intriguing outcome of the current study was the substantial increases in total energy of sorghum biomass from the *SbCCoAOMT* overexpression lines compared to wild-type without any observable negative impacts on plant growth or changes to lignin content. The total energy levels in *SbCCoAOMT* biomass were elevated by 60–75 cal g^-1^, which represents a considerable increase in total energy per gram of biomass. Overexpression of *SbCCoAOMT* appears to increase the amount of energy captured and stored in sorghum biomass. Based on the energy levels of neutral and acid detergent washed stover, the increased energy observed in the CCoAOMT overexpression lines can be attributed to cell wall bound (esterified) phenolic compounds, whose linkages are resistant to neutral detergent, but not to acid detergent. Similarly, overexpression of the sorghum *SbMyb60* transcription factor led to the identification of one line (Myb*-*ZG-124-1-2a) whose *SbMyb60* expression level was only modestly elevated relative to wild-type. While ADL and Klason lignin levels did not differ between stover collected from Myb*-*ZG-124-1-2a and wild-type, this line had higher total energy levels compared to wild-type and to the rest of the *SbMyb60* lines included in the study, and the soluble and wall bound hydroxycinnamates (caffeic, ferulic and sinapic acids) were also elevated relative to wild-type in this line [52, 53]. The results from both the *SbCCoAOMT* and *SbMyb60* overexpression studies suggest that increasing levels of ester-linked phenolic compounds, such as ferulic and sinapic acids, contributes to energy levels in biomass. Although *SbCCoAOMT* overexpression increases the availability of both feruloyl and sinapoyl groups for esterification in the cell wall, no changes in G- or S- lignin were observed, which indicates that the induction of additional pathway steps are necessary to incorporate these subunits into the lignin polymer.

RNA-seq analysis allowed for a comprehensive view of how overexpression of *35S*::*SbCCoAOMT* impacted both global gene expression and the expression levels of genes coding monolignol biosynthetic enzymes in sorghum. Overall, overexpression of *SbCCoAOMT* did not affect the expression of genes encoding monolignol biosynthetic enzymes, except for *SbCCoAOMT* (Sobic10G052200.1), whose elevated transcript levels also resulted in the accumulation of the corresponding protein in both transgenic lines (Figs 2B and 8). The expression levels of PAL, which catalyzes the first step of monolignol biosynthesis pathway, was significantly up-regulated only in *SbCCoAOMT*-9a leaf and stalk tissues. In contrast to our study, repression of *CCoAOMT* in petunia, via RNAi, downregulated the expression of a *CCR* gene, whose gene product catalyzes next step in the monolignol biosynthesis following CCoAOMT [68]. In general, these results show that altering expression of *CCoAOMT* causes only minor perturbations to expression levels of genes encoding monolignol biosynthetic enzymes.

Although overexpression of *SbCCoAOMT* did not have a major impact on global gene expression in either stalks or leaves, the expression of genes encoding enzymes linked to cell wall biosynthesis were elevated in *SbCCoAOMT* lines and correlates with patterns of gene-expression network analyses. Expression levels of a DAHP synthase (Sobic.001G351000) and a shikimate kinase (Sobic.006G235500), which both encode enzymes in aromatic amino acid synthesis [69], were also elevated in the two transgenic lines compared to wild-type in stalk tissues. Higher expression of DAHP synthase and shikimate kinase transcripts and higher levels of shikimate quantified through metabolite profiling indicate that *SbCCoAOMT* overexpression may stimulate flux through the shikimate pathway, which ultimately lead to the synthesis of aromatic amino acids required for the monolignol biosynthesis [31]. Although DEG analysis presented relatively few genes associated with cell wall biosynthesis, WGNCA analysis corroborated patterns of genes up-regulated in stalk tissue for both transgenic events that were related to pentose phosphate pathway, aromatic amino acid biosynthesis and phenylpropanoid biosynthesis. Hence, *SbCCoAOMT* overexpression may result increases in the substrate and cofactors required monolignol pathway.

Utilization of biomass as a carbon-neutral resource has received increased attention due to its availability, sustainability, renewability and energy security [8, 70]. Thermochemical conversion of biomass spans a range of technologies, which includes pyrolysis, gasification and direct combustion. Pyrolysis is the thermal decomposition of lignocellulosic biomass, and leads to valuable products including char, liquid (tar and oil) and gas products (syngas) [71]. We demonstrate overexpression of CCoAOMT can increase the energy density of biomass, which may be beneficial to thermoconversion processes. Alternatively, ferulic acid is a potential valuable co-product from the hydrolysis of biomass to produce sugars from cell wall polysaccharides, and it has a wide range of biomedical applications that include antioxidant, anti-inflammatory, UV-protectant and anti-microbial activities with potential usage in nutraceutical, cosmetic and food products [72].

In conclusion, this study demonstrates that *SbCCoAOMT* can be manipulated to modify cell wall composition of soluble and wall-bound phenolic compounds without significant negative impacts on plant growth and development and minimal impacts on other biochemical pathways. Moreover, this study indicated that overexpression of *SbCCoAOMT* increased levels of phenolic compounds derived from the monolignol biosynthesis pathway, which may have potential applications in the emerging bioenergy sector.

## Supporting information

S1 Fig Overexpression of *SbCCoAOMT* in wild-type sorghum. Immunoblot detection of CCoAOMT from leaves (top) and stalks (bottom). Protein extracts from wild-type were separated by SDS-PAGE, transferred to membrane, and probed with polyclonal antibodies raised against the recombinant SbCCoAOMT protein. Monoclonal antibodies raised against actin protein were used as a protein loading control. The exposure was increased to detect the presence of the CCoAOMT protein in wild-type extracts.
(TIF)

S2 Fig *SbCCoAOMT* overexpression induced changes in cell wall composition in leaf midrib from wild-type and *SbCCoAOMT* transgenic plants (indicated on top of each image). Autofluorescence observed with a Nikon A1R confocal laser scanning microscope. Scale bar = 200 μm.
(TIF)

S3 Fig Impact of *SbCCoAOMT* overexpression on monolignol biosynthesis pathway genes. Global expression of monolignol biosynthesis genes were quantified from RNA-seq dataset: Phenylalanine ammonia lyase (PAL; Sobic.004G220300.1), cinnamate-4-hydroxylase (C4H; Sobic.002G126600.1), 4-coumarate-CoA ligase (4CL; Sobic.004G062500.1), hydroxycinnamoyl CoA:shikimate hydroxylase (HCT; Sobic.004G212300.1), *p*-coumarate-3-hydroxylase (C3H; Sobic.009G181800.1), cinnamyl CoA reductase (CCR; Sobic.007G141200.1), ferulate-5-hydroxylase (F5H; Sobic.001G196300.1), caffeic acid *O-*methyltransferase (COMT; Sobic.007G047300.1) and cinnamyl alcohol dehydrogenase (CAD; Sobic.004G071000.1). Asterisks indicate levels of significance for differential expression of *SbCCoAOMT* transgenic event compared to wild-type determined using DESeq2 (FDR: ** *p* ≤ 0.01).
(TIF)

S4 Fig Expression profiles for all 16 weighted gene co-expression network analysis (WGCNA) identified from sorghum stalks and leaves. Expression profiles of 16 co-expression modules were obtained from WGCNA.
(TIF)

S5 Fig Sparse partial least squares-discriminant analysis (sPLS-DA) on metabolite data from LC-MS negative and positive mode. (A) Leaf negative, (B) leaf positive, (C) stalk negative and (D) stalk positive mode with the top ranked 20 metabolites.
(TIF)

S1 Table Summary of paired-end Illumina reads and mapping results using HISAT2. ^a^ Post processing ^b^ Number inside parentheses indicate the percentage of total reads.
(XLSX)

S2 Table Analysis of metabolites from wild-type (RTx430), SbCCoAOMT-9a and SbCCoAOMT-28b biomass using LC/MS positive and negative mode. Values represent mean and ± 1 SE. Values in bold text indicate those that were statistically significantly different (p ≤0.05).
(XLSX)

S3 Table Analysis of soluble and wall-bound phenolics from wild-type (RTx430), SbCCoAOMT-9a and SbCCoAOMT-28b biomass using GC-MS. Values represent peak area of major ion and ± 1 SE (10^4). Values in bold text indicate those that were statistically significantly different (p ≤0.05).
(XLSX)

S4 Table Differentially expressed genes for SbCCoAOMT-9a and SbCCoAOMT-28b in leaf and stalk tissue compared to wild-type. Normalized counts, venn diagram designation, and log fold change (LFC) with associated p-value (FDR p<0.05).
(XLSX)
