# Supplementary figures and images for: Overexpression of the Sorghum bicolor SbCCoAOMT alters cell wall associated hydroxycinnamoyl groups
Source: PLoS One. 2018 Oct 5;13(10):e0204153. doi: 10.1371/journal.pone.0204153 (PMC6173380; doi:10.1371/journal.pone.0204153)

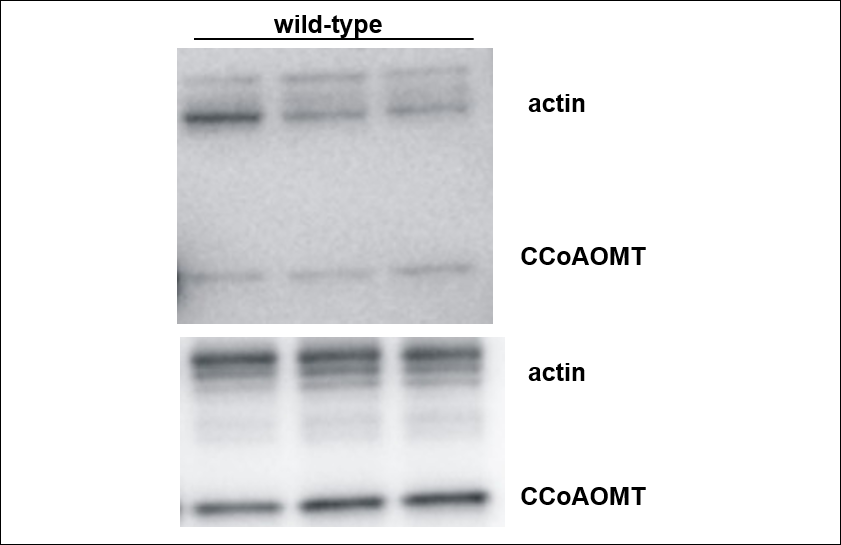

Supplement: S1 Fig — Immunoblot detection of CCoAOMT from leaves (top) and stalks (bottom). Protein extracts from wild-type were separated by SDS-PAGE, transferred to membrane, and probed with polyclonal antibodies raised against the recombinant SbCCoAOMT protein. Monoclonal antibodies raised against actin protein were used as a protein loading control. The exposure was increased to detect the presence of the CCoAOMT protein in wild-type extracts. (TIF) [file pone.0204153.s001.tif]

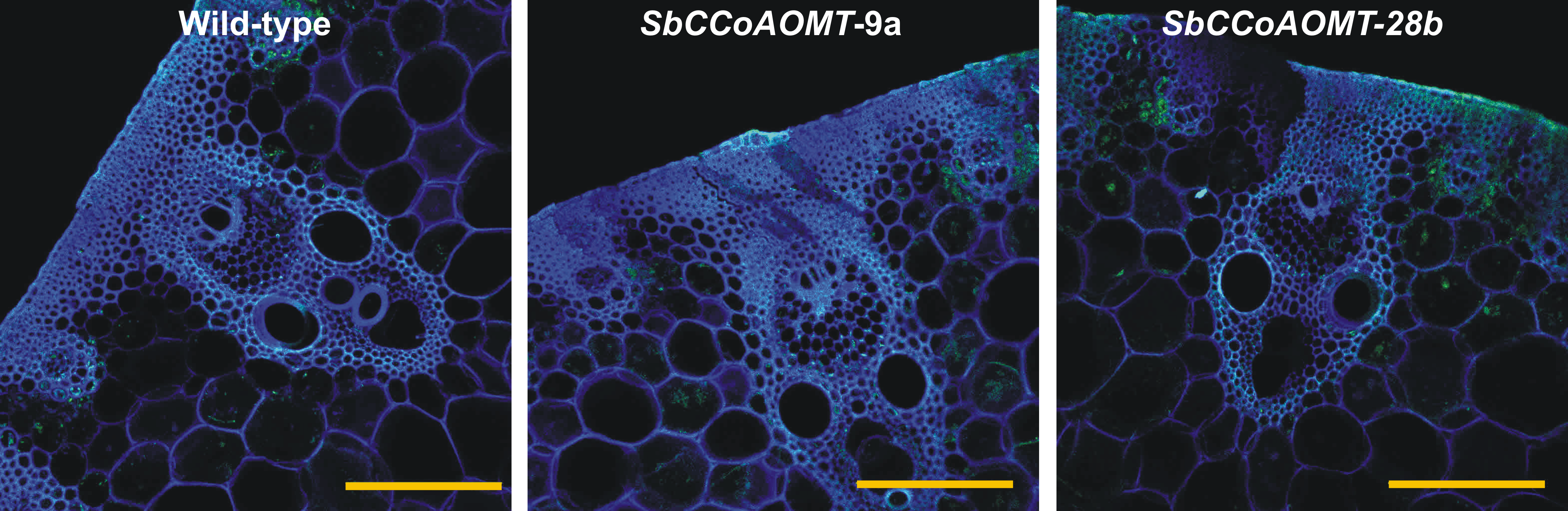

Supplement: S2 Fig — Autofluorescence observed with a Nikon A1R confocal laser scanning microscope. Scale bar = 200 μm. (TIF) [file pone.0204153.s002.tif]

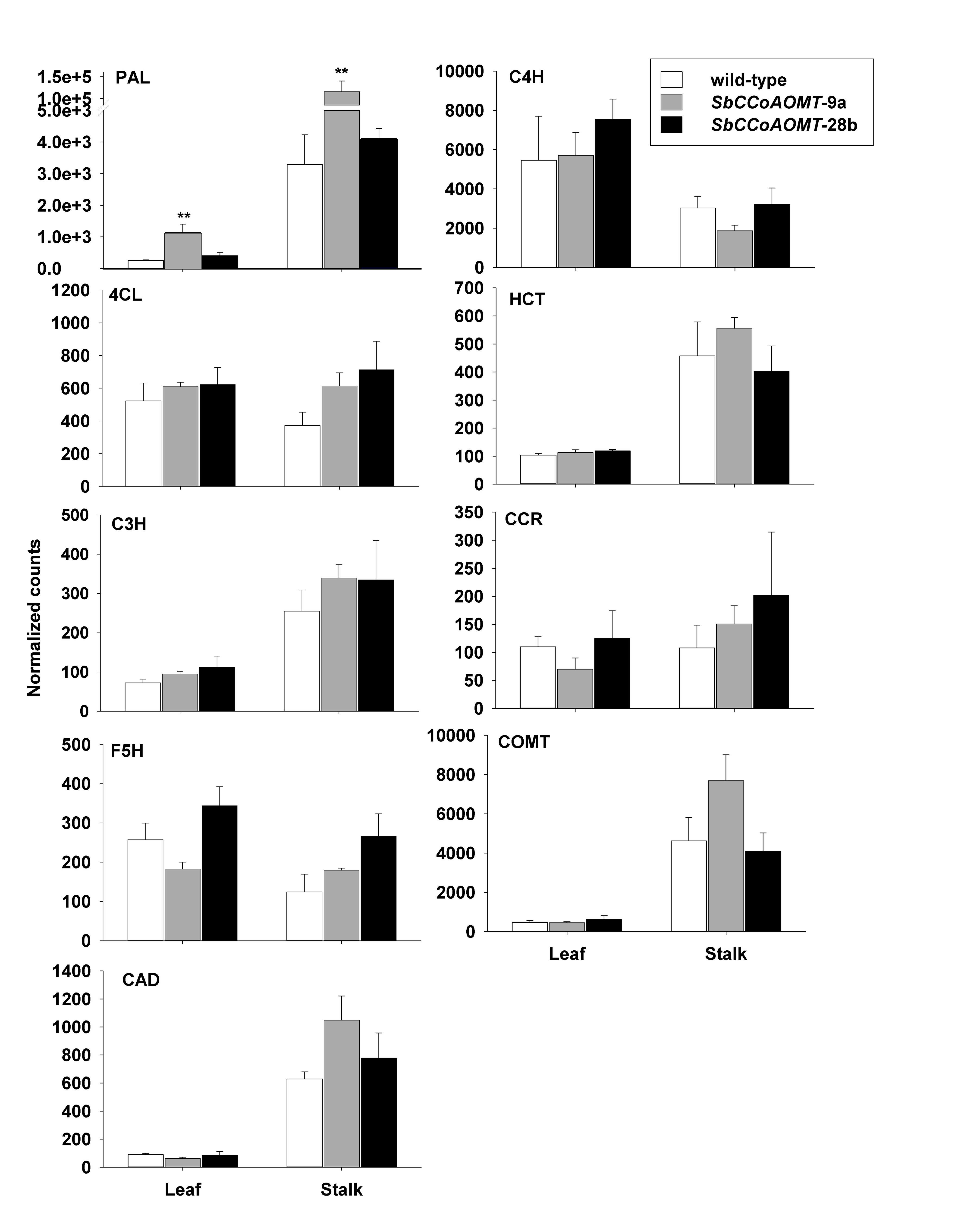

Supplement: S3 Fig — Global expression of monolignol biosynthesis genes were quantified from RNA-seq dataset: Phenylalanine ammonia lyase (PAL; Sobic.004G220300.1), cinnamate-4-hydroxylase (C4H; Sobic.002G126600.1), 4-coumarate-CoA ligase (4CL; Sobic.004G062500.1), hydroxycinnamoyl CoA:shikimate hydroxylase (HCT; Sobic.004G212300.1), p-coumarate-3-hydroxylase (C3H; Sobic.009G181800.1), cinnamyl CoA reductase (CCR; Sobic.007G141200.1), ferulate-5-hydroxylase (F5H; Sobic.001G196300.1), caffeic acid O-methyltransferase (COMT; Sobic.007G047300.1) and cinnamyl alcohol dehydrogenase (CAD; Sobic.004G071000.1). Asterisks indicate levels of significance for differential expression of SbCCoAOMT transgenic event compared to wild-type determined using DESeq2 (FDR: ** p ≤ 0.01). (TIF) [file pone.0204153.s003.tif]

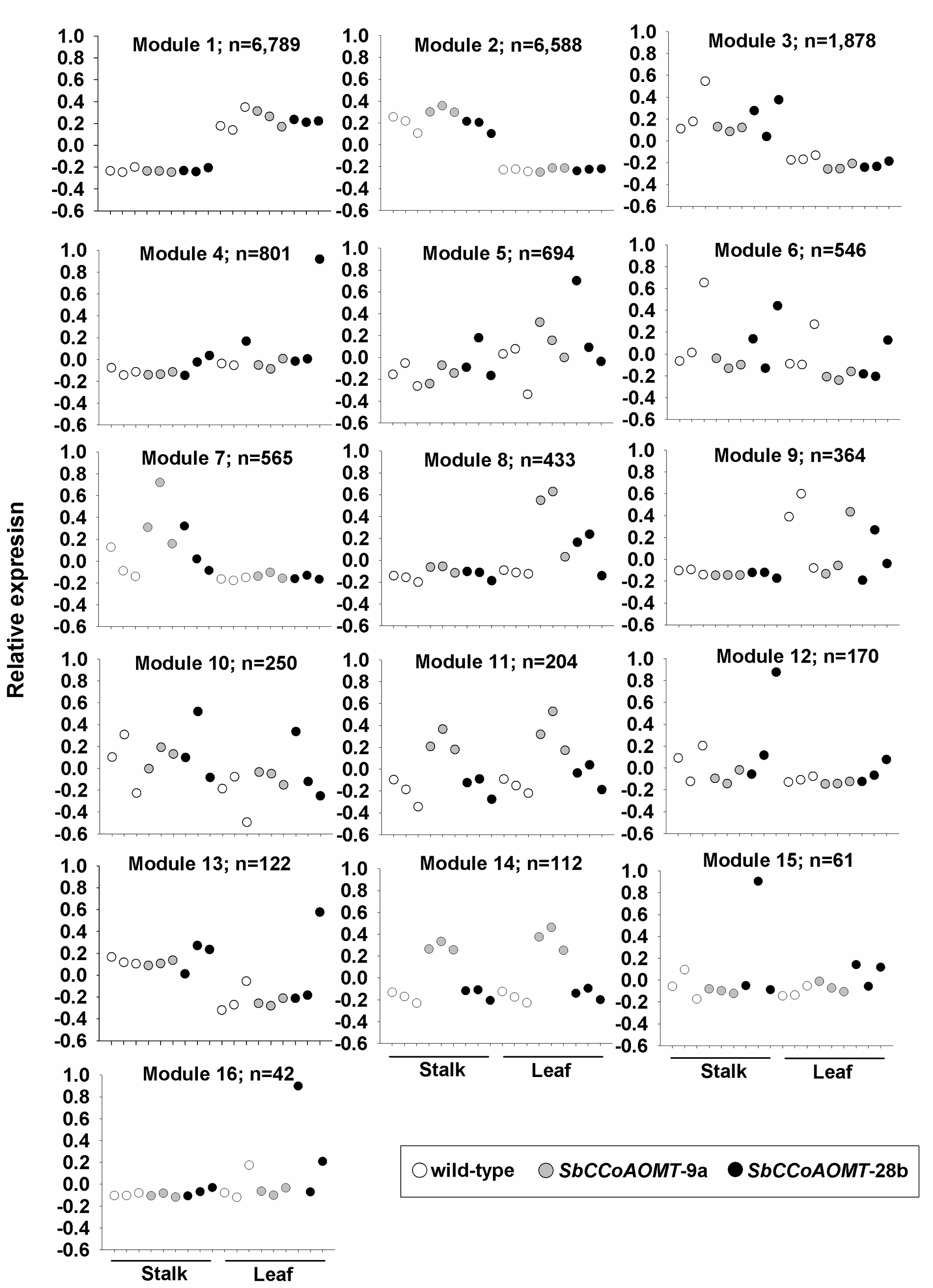

Supplement: S4 Fig — Expression profiles of 16 co-expression modules were obtained from WGCNA. (TIF) [file pone.0204153.s004.tif]

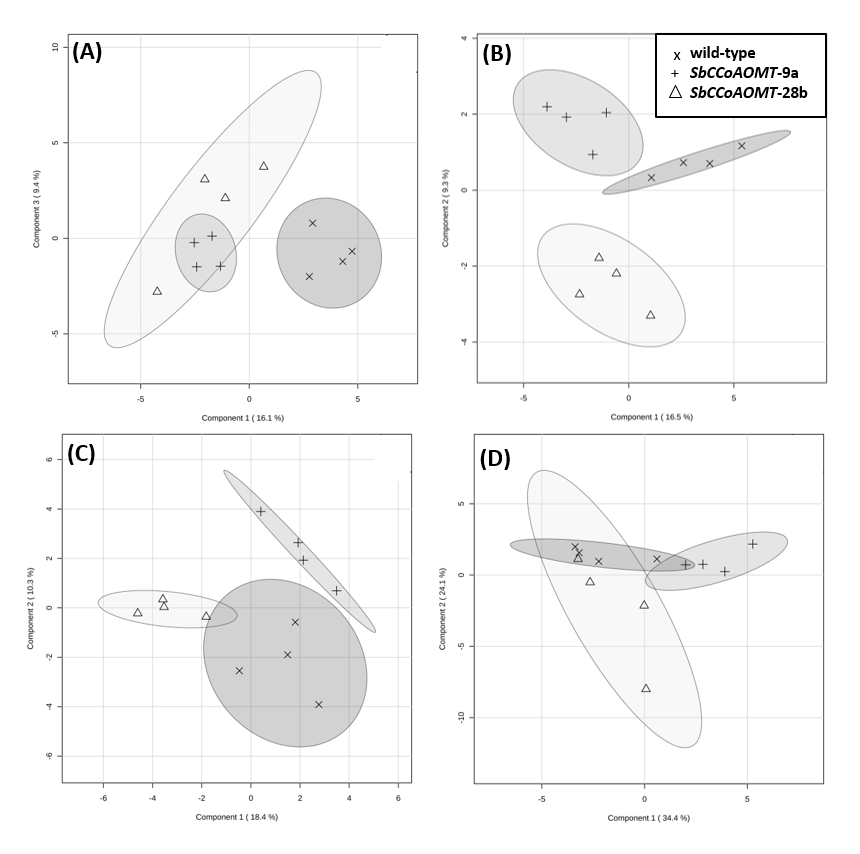

Supplement: S5 Fig — (A) Leaf negative, (B) leaf positive, (C) stalk negative and (D) stalk positive mode with the top ranked 20 metabolites. (TIF) [file pone.0204153.s005.tif]
